# Effectiveness of an add-on guided internet-based emotion regulation training (E-TRAIN) in adolescents with depressive and/or anxiety disorders: study protocol for a multicenter randomized controlled trial

**DOI:** 10.1186/s12888-022-04291-6

**Published:** 2022-10-14

**Authors:** Julie Emmelkamp, Marike A Wisman, Nico JM Beuk, Yvonne AJ Stikkelbroek, Maaike H Nauta, Jack JM Dekker, Carolien Christ

**Affiliations:** 1 Department of Youth and Family, Arkin Mental Health Care, Amsterdam, The Netherlands; 2 Department of Research, Arkin Mental Health Care, Amsterdam, The Netherlands; 3 Department of Clinical, Neuro- and Developmental Psychology, Vrije Universiteit Amsterdam, Amsterdam Public Health Research Institute, Amsterdam, The Netherlands; 4 Department of Clinical Child and Family Studies, Faculty of Social and Behavioral Sciences, Utrecht University, Utrecht, The Netherlands; 5 GGZ Oost Brabant, P.O. Box 3, 5427 ZG Boekel, The Netherlands; 6 Department of Clinical Psychology and Experimental Psychopathology, University of Groningen, Groningen, The Netherlands; 7 Accare Child Study Center, P.O. Box 660, 9700 RB Groningen, the Netherlands; 8 Department of Clinical Psychology, Vrije Universiteit Amsterdam, Amsterdam, The Netherlands; 9 Department of Research and Innovation, GGZ inGeest Specialized Mental Health Care, Amsterdam, The Netherlands; 10 Amsterdam UMC, Department of Psychiatry, Amsterdam Public Health Research Institute, VU University Medical Center, Amsterdam, The Netherlands

**Keywords:** Emotion regulation, Depression, Anxiety, Adolescents, E-mental Health, Internet-based intervention, Randomized controlled trial

## Abstract

**Background:**

During adolescence, depressive and anxiety disorders are among the most common mental health disorders. Both disorders tend to persist, are predictive for other mental disorders, and are associated with severe impairment in diverse areas. Although Cognitive Behavioral Therapy (CBT) has proven to be an effective treatment, a considerable number of adolescents do not respond to CBT and residual symptoms often remain. Therefore, it is of great importance to improve treatment outcomes for depressed and/or anxious adolescents. Dysfunctional emotion regulation appears to be a transdiagnostic factor in the development and maintenance of aforementioned disorders. Enhancing emotion regulation skills may therefore reduce symptom severity. In light of this, we developed a guided internet-based emotion regulation training (E-TRAIN) that will be added to CBT. This study aims to evaluate the effectiveness of E-TRAIN + CBT compared to CBT alone on depressive and anxiety outcomes among adolescents with depressive and/or anxiety disorder.

**Methods:**

In this multicenter two-arm randomized controlled trial with parallel group design, we aim to include 138 adolescents, aged 13–19 years, referred for treatment and diagnosed with depressive and/or anxiety disorder. Participants will be allocated to either CBT or CBT + E-TRAIN. Assessments will take place at baseline, and at 3 (T1), 6 (T2) and 12 (T3) months after baseline. We will conduct multi-informant assessments: the adolescent, a parent/caregiver, and the CBT therapist will be asked to fill in questionnaires. The continuous primary outcome measure is self-reported depressive and anxiety symptoms at six months after baseline, measured with the RCADS25. Secondary outcome measures include anxiety or depression diagnosis based on a semi-structured clinical interview, emotion (dys) regulation, and parent-report measures of anxiety, depression and emotion (dys) regulation.

**Discussion:**

This study is the first randomized controlled trial to examine the additional value of a guided internet-based emotion regulation training to regular CBT in adolescents with depressive and/or anxiety disorders. If this intervention is effective, it can be implemented in mental health care and improve treatment for these young people.

**Trial registration:**

Registered on June 23, 2021 in The Netherlands Trial Register (NL9564). Retrospectively registered. Recruitment started in May 2021 and is ongoing.

**Supplementary Information:**

The online version contains supplementary material available at 10.1186/s12888-022-04291-6.

## Background

During adolescence, depressive and anxiety disorders are among the most common mental disorders (e.g., [1, 2]), and they frequently co-occur [3]. Prevalence rates are reported to increase significantly during adolescence and are estimated between 3–7% for depressive and between 6–15% for anxiety disorders [1, 2]. Both types of disorder are associated with various adverse consequences for social, academic, and family functioning [e.g., [4, 5]]. Additionally, childhood anxiety and depressive disorders tend to persist into adulthood and predict a range of other mental health disorders in adulthood, including bipolar disorders and substance abuse (e.g., [4, 6–11]). Moreover, both disorders are associated with an increased risk of suicide [12]. Suicide is the leading cause of death in adolescents (aged 15–24) in Europe, and the fourth leading cause in adolescents (aged 15–19) worldwide [13, 14].

Adolescence marks the transition from childhood into adulthood and is a crucial period for the development of mental disorders [15, 16]. The life stage of adolescence is characterized by substantial biological and socio-emotional changes (e.g., [17, 18]). Due to these changes and new challenging life tasks (e.g., academic challenges, instable peer and romantic relationships), adolescents often experience increased emotionality [19–21]. Thus, new ways of adequately responding to emotions need to be established in this period. Additionally, adolescence is characterized by an increasing autonomy and decreasing dependence on parental regulation, resulting in a more independent and internal management of emotions (e.g., [19, 22]). However, evidence suggests that adolescents use more maladaptive strategies and less adaptive strategies to cope with emotions than younger children [23]. The substantial changes and life tasks during adolescence in combination with the increasing demands on their autonomy, leaves adolescents at specific risk for emotion regulation difficulties [23].

Emotion regulation (ER) is defined as “the processes responsible for monitoring, evaluating, and modifying emotional reactions, especially their intensive and temporal features, to accomplish one’s goals” ([24], p. 27–28). Difficulties in ER are associated with various mental health problems, including anxiety and depression [19, 25–27]. Schäfer et al. [26] showed in a meta-analysis that maladaptive ER strategies (i.e., avoidance, suppression, and rumination) were associated with more depressive and anxiety symptoms in non-clinical samples of adolescents, whereas adaptive ER strategies (i.e., cognitive reappraisal, problem solving, and acceptance) were related to less depressive and anxiety symptoms. Correspondingly, children and adolescents with depressive and anxiety disorders tend to use more maladaptive strategies and less adaptive strategies (i.e., more withdrawal, and less reappraisal) than those without these disorders [28–30]. By applying adaptive ER strategies adolescents are able to handle emotional challenges, and these adaptive strategies may therefore be a protective factor against psychopathology [31]. A recent meta-analysis showed that improvements in ER skills were associated with reduced depression and anxiety in adolescents aged 14–24 [32]. To date, however, even though interventions exist that may improve ER skills for these adolescents (e.g., traditional CBT, dialectical behavior therapy, mindfulness-based cognitive therapy), most of them do not exclusively focus on targeting ER [33, 34].

For adolescents with depressive and anxiety disorders, Cognitive Behavioral Therapy (CBT) is considered the treatment of choice [35, 36], with medium to large effect sizes in symptoms reduction [37]. However, its mean effect compared to usual care is only modest [38], a considerable number of adolescents do not respond to CBT, or still have residual symptoms, that may – in turn – put them at risk for relapse [37, 39]. Given the high prevalence and undesirable effects of anxiety and depressive disorders in adolescents, it is of great importance to improve treatment outcomes of them.

Since ER seems to be an important transdiagnostic factor across depressive and anxiety disorder [26], enhancing ER might be a promising target in the treatment of these disorders. In an RCT among depressed adults, adding an emotion regulation training to CBT resulted in a significant decrease in depressive symptoms compared to CBT alone [40]. Little research has been conducted on ER interventions in adolescent samples with anxiety and depressive disorders. Holmqvist Larsson et al. [41] showed that a group skills training in ER for adolescents and their parents led to a decrease in ER difficulties in an outpatient sample of adolescents with a mental disorder. Furthermore, in a recent review on emotion regulation focused interventions four studies that include adolescents with internalizing disorders are discussed [34]. In all four studies, adding an ER regulation component to the intervention led to improvement of ER skills in adolescents [42–45].

We developed a guided internet-based emotion regulation training called *E-TRAIN* that will be added to Cognitive Behavioral Therapy (CBT). Internet-based treatments may provide an accessible and feasible alternative to face-to-face treatments: they fit to the digital lifestyle of adolescents, are less time-consuming, and show advantages in terms of convenience [46, 47]. Moreover, numerous meta-analyses have demonstrated that internet-based therapy is effective in treating depressive and anxiety disorders in children and adolescents [48–51]. We decided to create a guided intervention because there is meta-analytical support of the superiority of guided internet-based treatments to unguided treatments in terms of efficacy and acceptability in adults [46, 52, 53]. For adolescents with anxiety and/or depression, a recent meta-analysis showed that guided internet-based treatments (of at least 90 min) were superior to unguided treatments [50].

### Research aims

The current study aims to evaluate the clinical effectiveness of the addition of a guided internet-based ER training (E-TRAIN) to Cognitive Behavioral Therapy (CBT) in reducing depressive and anxiety symptoms in adolescent patients (aged 13–19) with depressive and anxiety disorders. Second, this study aims to examine the effectiveness of E-TRAIN added to CBT in enhancing ER among adolescents. The third study aim is to examine the acceptability and feasibility of this internet-based guided emotion regulation training.

## Methods

### Design

**Fig. 1:**
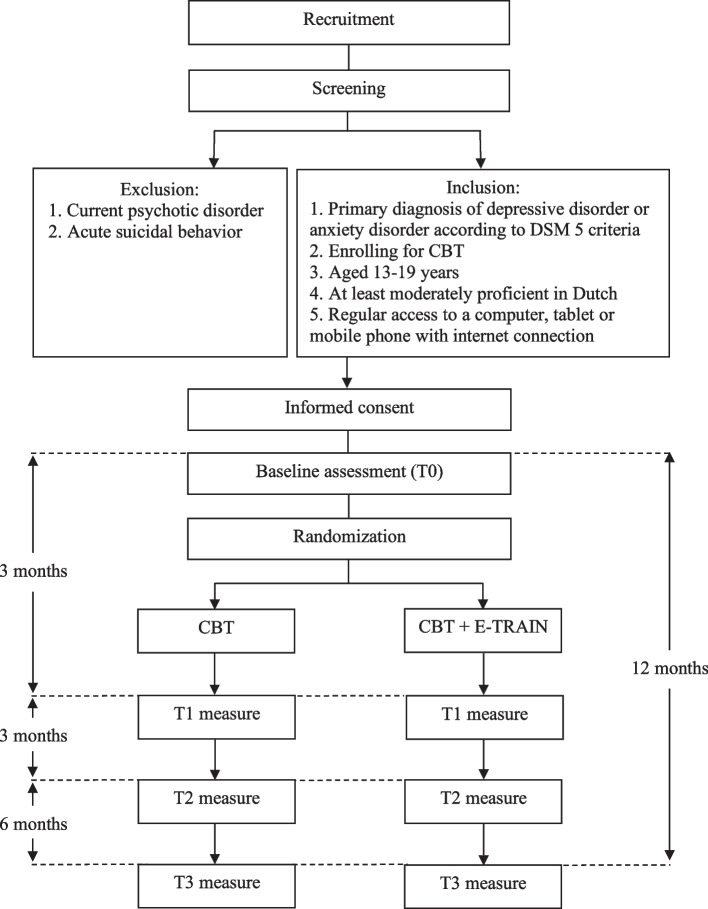
Trial flowchart

To determine the effectiveness of adding E-TRAIN to CBT, we will conduct a multicenter two-arm randomized controlled trial (RCT) using a two-arm parallel group design. We aim to include 138 adolescents with a primary diagnosis of depressive and/or anxiety disorder. After completion of baseline assessment (T0), participants will be allocated to either CBT or CBT + E-TRAIN. Follow-up assessments will take place after 3 (T1), 6 (T2: primary outcome), and 12 months (T3) post-baseline assessment. See Fig. 1 for the trial flowchart. All assessments are online questionnaires, which will be completed by multiple informants (adolescents, parents/caregivers, and therapists), except for the Mini (KID) International Neuropsychiatric Interview (MINI version 7.02) [54, 55], which will be assessed in a telephone interview 6 months after baseline. This protocol was written in accordance with the Standard Protocol Items: Recommendations for Interventional Trials (SPIRIT) guideline [56]. This study has been registered in the Netherlands Trial Register (NL9564). The Medical Research Ethics Committees United (MEC-U) in the Netherlands has approved the study protocol.

### Participants

The target population consists of adolescents who have been referred to a mental health center and have been diagnosed with depressive disorder and/or anxiety disorder. In order to be eligible, a participant must meet all of the following criteria: 1) primary diagnosis of depressive disorder and/or anxiety disorder according to DSM-5 criteria; 2) enrolling for CBT in regular care; 3) aged 13–19 years; 4) at least moderately proficient in Dutch; and 5) regular access to a computer, tablet or mobile phone with internet connection. Participants will be excluded from participation in this study if they display a current psychotic disorder or acute suicidal behavior.

### Sample size

We will conduct a pairwise comparison between the treatment arms (CBT + E-TRAIN vs. CBT). The continuous primary outcome measures will be depressive and anxiety symptoms at T2. A priori sample size calculation was performed using the *sampsi* command in Stata (version 14.0; Stata Corp, College Station, USA). We aim to include *N* = 138 adolescents (*n* = 69 in each group) to be able to detect a Cohen’s d of 0.40 with α = 0.05 (two-sided) and power = 0.80, taking into account 35% extra inclusion to account for drop-out.

### Procedure

#### Recruitment and consent

Participants will be recruited at the youth departments of Arkin and GGZ Oost Brabant, two Dutch mental health centres. Other additional treatment sites may be added. All adolescents will be screened for eligibility during the intake by a clinician, who assesses inclusion and exclusion criteria. Diagnoses will be established by a psychiatrist or a registered Health Care Psychologist (Dutch: "Gezondheidszorg-psycholoog") on DSM-5 criteria. After the intake, eligible adolescents and their parents or caregivers will receive written study information and the informed consent forms. If the eligible adolescents agree to be approached, a research assistant will contact them by telephone and provide further information about participation in the study. If adolescents agree to participate in the study, they will be asked to sign the informed consent form. Both parents will be asked to sign the informed consent form for their child’s participation if the patient is < 16 years. Additionally, one of the parents will be asked to participate in the study and fill in questionnaires as well. This parent will also be asked to sign an informed consent form regarding their own participation.

#### Assessments

After signing informed consent, all participants will be invited to fill in the baseline assessment (T0). Adolescents and their parents will receive an email with a link to the questionnaires. All assessments will be conducted via the internet, using Castor EDC (Castor Electronic Data Capture (2021.2)). Those who have not completed the assessment within five days will receive a reminder via email. After one week, participants who have not completed the assessment will be approached via telephone by a research assistant. If necessary, adolescents will have the opportunity to fill in the baseline assessment in the presence of a research assistant, who can provide guidance. After completing baseline assessment, participants will receive a voucher of 15 euros.

Follow-up assessments will be administered at 3 months, 6 months, and 12 months after baseline. Similar to the procedure for baseline assessments, participants (adolescents and parents/caregivers) who have not completed the assessment after one week will be reminded via email and telephone. After completing follow-up assessments, participants (adolescents and parents) will receive a voucher of 10 euros for each completed T1 assessment, and 15 euros for each completed T2 and T3 assessment. Additionally, after completion of the T2 assessment, a trained research assistant who is blind for treatment condition will contact each adolescent for a short diagnostic interview by phone to determine the presence of depressive and anxiety disorders at 6-month follow-up. The depression and anxiety sections of the MINI (KID) will be used. After completion of the MINI (KID), each participant will receive a voucher of 5 euros. Both the E-TRAIN and the CBT therapist of the adolescent will fill in short questionnaires as well. The CBT therapist will be asked to complete questionnaires at T0, T1, and T2, and the E-TRAIN therapist will fill in a questionnaire after completion of E-TRAIN.

#### Randomization

After baseline assessment has been completed by the adolescent, randomization will be carried out. Randomization of participants will be performed via Castor EDC (Castor Electronic Data Capture (2021.2)) by a researcher who is blind to block size and order. Randomization will take place at an individual level, stratified by primary diagnosis (depressive or anxiety disorder) and treatment site using a computer-generated block randomization schedule. To ensure that an equal number of patients are allocated to CBT and CBT + E-TRAIN, the allocation ratio will be 1:1. To prevent selection bias, all researchers will be blind to random block order. No researchers will have access to the randomization schedule. Due to the nature of the add-on design, blinding of adolescents, parents, therapists, and researchers to treatment condition is not applicable. The research assistants assessing the diagnostic interview at T2 will be blinded to treatment condition.

#### Focus groups

Additionally, we will organize two focus groups to evaluate the experimental intervention. We aim to include six randomly selected participants for each focus group. Adolescents who participate in the focus group will receive a voucher of 25 euros. Focus groups will take approximately 1.5 h, and will be led by a psychologist and a researcher. In addition, we will organize a focus group with a random sample of six E-TRAIN therapists to examine the acceptability of the internet-based emotion regulation training.

### Interventions

#### E-TRAIN

Adolescents allocated to the experimental condition will partake in a guided internet-based emotion regulation training (E-TRAIN), that was developed for the purpose of the current study. E-TRAIN is based on existing face-to-face emotion regulation interventions for children and adolescents [57, 58], adults [33], and a recently developed internet-based emotion regulation training for depressed adults [59]. In order to develop an internet-based module tailored for adolescents, we first performed a pilot feasibility study using a brief, existing internet-based emotion regulation training module [60] among our target population at the youth mental health care department of Arkin. We conducted focus groups with pilot participants (*N* = 5) and youth therapists (*N* = 6) aimed at 1) evaluating the brief emotion regulation module; 2) identifying key strategies for improvement; and 3) identifying adolescents’ preferences and needs regarding internet-based treatment. Based on the existing emotion regulation interventions and the conclusions provided by these focus groups, we developed a first version of the E-TRAIN module. Subsequently, this version was tested and evaluated by a small sample of adolescent patients with anxiety and/or depression (*N* = 4), after which we further adapted and finalized the E-TRAIN module.

**Fig. 2:**
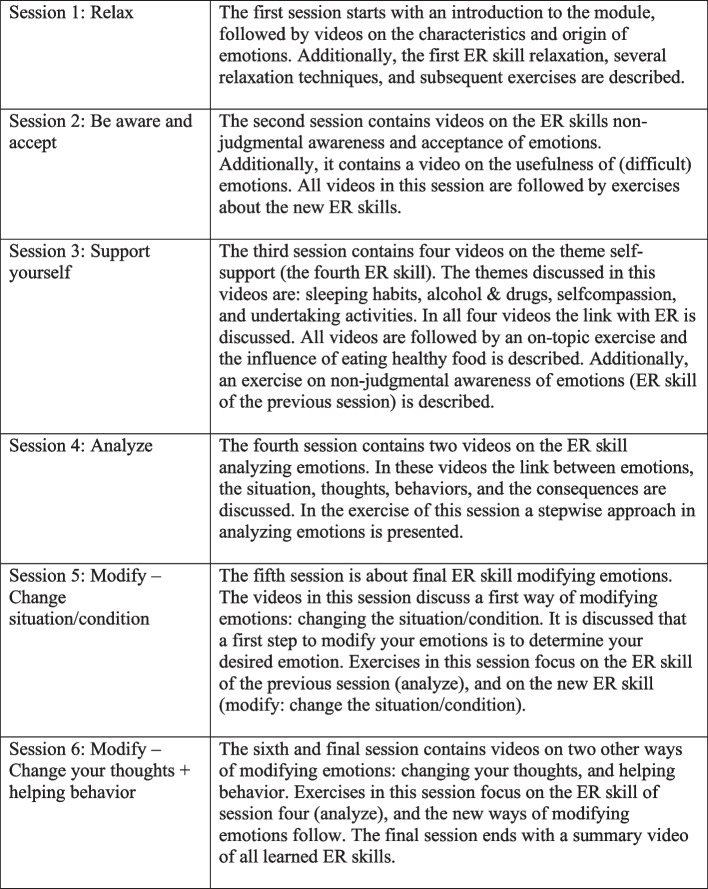
Summary of E-TRAIN sessions

E-TRAIN will be provided on a secured online platform (*Jouw Omgeving*). It consists of six sessions in which six ER skills are covered: 1) relaxation; 2) non-judgmental awareness of emotions; 3) acceptance of emotions; 4) self-support; 5) analyzing emotions; and 6) modifying emotions. Adolescents receive psycho-education about these skills, and about the origin and functions of emotions in animated videos (of 1–5 min). Moreover, each session includes short written texts, exercises, relevant examples, patient stories, and audio files. By listening to these audio files, the adolescent can practice with the newly acquired ER skill and previously acquired skills. After each session, the adolescent is stimulated to listen to this audio file at least three more times before the next session as a homework assignment. See Fig. 2 for more detailed information on each session. Each E-TRAIN session will take approximately 25 min to complete. After each session a trained therapist will provide feedback on the exercises, using a secured chat function within the online platform, to guide the adolescent in correctly applying the learned ER skills.

Additional to these six online sessions, E-TRAIN consists of three video conference sessions with a therapist. At the start of E-TRAIN, a video conference session of 30 min will be scheduled. This video conference session will be held in order to acquaint patients with their therapist and vice versa, and to determine E-TRAIN treatment goals. After finishing the first three online sessions, a second video conference session will be scheduled to provide guidance and monitor the patient's progress. At the end of E-TRAIN, a final video conference session will be scheduled. In this session, the treatment goals will be evaluated and the focus will be on applying the learned emotion regulation skills in daily life.

#### E-TRAIN therapists

Patients will be guided by a psychologist who has been trained in the application of E-TRAIN. After each online session, these E-TRAIN therapists will provide feedback and guidance using secured messages within the online platform. To ensure treatment integrity, E-TRAIN therapists will receive a detailed treatment protocol with standardized feedback templates. E-TRAIN will take place simultaneously to CBT, and will start after the first 2–4 CBT sessions. Based on the patient’s preference, the six sessions of E-TRAIN will be completed on either a weekly or fortnightly schedule; hence, E-TRAIN will be completed in 6 to 12 weeks. All adolescents will be guided by an E-TRAIN therapist other than their CBT therapist.

#### Treatment as usual: CBT

Both groups will receive CBT, a commonly used evidence-based therapy for adolescents with anxiety [61] and depression [62, 63]. CBT will be provided by experienced MSc or registered Health Care psychologists. The primary goal of CBT is to identify and restructure maladaptive thought patterns and behaviors (e.g., [64, 65]). Key components of CBT for adolescents with depressive and anxiety disorders are 1) psychoeducation; 2) behavioral activation, exposure, and behavioral experiments; 3) cognitive restructuring; and 4) relapse prevention [65, 66].

### Outcome measures

#### Primary outcome measure

Depressive and anxiety symptoms as a unidimensional construct will be measured with the child self-report version of the Revised Children’s Anxiety and Depression Scale – Short Version (RCADS25) [67]. This questionnaire is recommended by the International Consortium for Health Outcomes Measurement (ICHOM) as a questionnaire for measuring anxiety and depression in children and adolescents [68]. The RCADS25 assesses anxiety and depression according to the DSM-IV and consists of two subscales: the 15-item anxiety subscale and the 10-item depression subscale [67]. The total score on the RCADS 25 will be used. The questionnaire has shown a good internal consistency (α = 0.89) (Bagheri et al., 2019), and acceptable reliability and validity [67].

#### Key secondary outcome measures

See Table 1 for an overview of all measurement instruments and timepoints for adolescents, parents/caregivers, and therapists.

**Table 1:** Overview of instruments per assessment

**Measures**	**T0**	**T1**	**T2**	**T3**^**a**^
*Patient*				
Demographics	x			
RCADS25	x	x	x	x
CDI-2	x	x	x	x
SCARED	x	x	x	x
FEEL-KJ	x	x	x	x
DERS	x	x	x	x
AUDIT	x	x	x	x
SRSQ	x	x	x	x
IPPA short form	x		x	
RSES	x		x	
Life Events	x			x
COVID-19		x	x	x
CSQ-8			x^b^	
*Parent*				
Demographics	x			
CDI-P	x	x	x	
SCARED-P	x	x	x	
FEEL-KJ-P	x	x	x	
DERS	x	x	x	
CCNES-A	x		x	
BSI-18	x			
*CBT therapist*				
CGI	x	x	x	
CGAS	x	x	x	
*E-TRAIN therapist*				
SUS			x	

^a ^T0: baseline, T1: 3 months after baseline, T2: 6 months after baseline, T3: 12 months after baseline

^b ^Will only be assessed in the E-TRAIN (experimental) group

##### Depressive symptoms

Depressive symptoms as a separate construct will be measured with the Children’s Depression Inventory (CDI-2; Dutch version) [69]. The 28-item CDI-2 is a revision of the CDI [70] that assesses affective, cognitive, and somatic symptoms of depression in youth (7 to 17 years) over the past two weeks [69]. All 28 items are rated on a 3-point Likert scale from 0 to 2, and scores range from 0 to 56. The CDI-2 shows good psychometric properties [71, 72], and has been validated in adolescents up to the age of 21 [69]. To obtain a multi-informant assessment of adolescents’ depressive symptoms, the parent version of the CDI-2 will be used to asses depressive symptoms according to the parent (CDI-P) [70]. The CDI-P comprises of 17 items rated on a 4-point Likert scale, and shows good psychometric qualities [72].

##### Anxiety symptoms

Anxiety symptoms as a separate construct will be assessed with the Dutch translation of the 69-item Screen for Child Anxiety Related Disorders (SCARED; Dutch version) [73, 74]. The SCARED-NL assesses anxiety disorders according to the DSM-IV in children aged 7 to 19 years [73]. All items are rated on a 3-point Likert scale. The SCARED has demonstrated a high internal consistency, good test–retest reliability, and adequate construct and predictive validity [74, 75]. Additionally, the SCARED – Parent Version will be used to assess anxiety symptoms according to the parent (SCARED-P) [74]. The SCARED-P consists of 69 items rated on a 3-point Likert scale, and shows good psychometrics [74].

##### Diagnosis of depression and anxiety

he presence of a current diagnosis of depression and/or anxiety at T2 will be assessed with the MINI (KID) (version 7.02) [55, 56]. The MINI is a structured, clinician-administered diagnostic interview that is widely used to assess the presence of psychiatric disorders based on the Diagnostic and Statistical Manual of Mental Disorders (Fifth edition; DSM-5) and the International Classification of Diseases (Tenth revision; ICD-10). For adolescents aged 13–17, sections A, B, D, E, F, G, H, U of the MINI KID will be administered. For adolescents aged 18 or 19 years, sections A, AY, B, E, D, F, FA, and N of the MINI will be administered. Both the MINI and MINI KID are well-validated, reliable interviews [54, 55, 76].

#####  Emotion regulation

Emotion regulation will be measured with two questionnaires: the Dutch translation of *Der Fragebogen zur Erhebung der Emotionsregulation bei Kindern und Jugendlichen* (FEEL-KJ: Duth version) [77, 78] and the Difficulties in Emotion Regulation Scale (DERS) [79]. The FEEL-KJ is a 90-item questionnaire that assesses 15 emotion regulation strategies (i.e., problem solving, distraction, forgetting, acceptance, humor enhancement, cognitive problem solving, revaluation, giving up, withdrawal, rumination, self-devaluation, aggressive actions, social support, expression, and emotional control) in response to anger, anxiety, and sadness in children and adolescents aged 10–19 years. All items are rated on a five-point Likert scale ranging from never to almost always. The primary emotion regulation strategies are divided into two secondary scales: the adaptive and maladaptive emotion regulation scale. Total scores on the secondary FEEL-KJ scales (adaptive and maladaptive scale) will be used. The FEEL-KJ is a reliable and valid instrument to measure emotion regulation in youth [77, 80].

The 36-item DERS assesses emotion regulation difficulties across six dimensions: non-acceptance of emotional responses, difficulty in engaging in goal-directed behavior, difficulty in controlling impulses, lack of emotional awareness, limited access to emotion regulation strategies, and lack of emotional clarity [79]. For each item, respondents rate how often they engage in different behaviors or have certain feelings on a 5-point Likert scale. Total DERS scores will be used, with higher DERS scores reflecting more emotion regulation difficulties [79]. The DERS was originally developed for adults, but has demonstrated high reliability and adequate validity for adolescents [81, 82] and is widely used in adolescent-research (e.g., [25]).

##### Global functioning, symptom severity and improvement

To asses global functioning of the adolescents, the CBT-therapist of the adolescent will fill in the Children’s Global Assessment Scale (CGAS) [83]. Adolescents are rated on a scale from 1 (extremely impaired) to 100 (superior functioning). To measure symptom severity and improvement the Clinical Global Impression Scales will be used (CGI) [84]. The CGI-Severity (CGI-S) is a 7-item scale ranging from a score of 1 (not ill) to 7 (requires inpatient care). The CGI-Improvement (CGI-I) is a 7-item scale that compares ratings of severity with previous measures, with scores ranging from 1 (very much improved) to 7 (very much worse). Both the CGAS and the CGI are widely used measures [83, 85].

#### Other variables of interest

##### Adolescent measures

In addition, other variables of interest that will be assessed in adolescents are:

- Socio-demographics, collected at baseline.
- Intervention uptake of CBT, registered by the CBT therapist.
- Perceived impact of COVID-19, as measured with a self-developed COVID-19 questionnaire. See supplementary file 1 for an English translation of this questionnaire.
- Alcohol use, as measured with the Alcohol Use Disorders Identification Test (AUDIT) [86].
- Life events, as measured with a self-developed Life Events Questionnaire. See supplementary file 2 for an English translation of this questionnaire. This questionnaire is based on the Clinician-Administered PTSD scale for children and adolescents (CAPS-CA) and the Life Events Checklist [87, 88].
- Quality of the parent–child relation, as measured with the parent scale of the Inventory of Parent and Peer Attachment short form (IPPA-s) [89, 90].
- Self-esteem, as measured with the Rosenberg Self-Esteem Scale (RSES) [91].
- Sleep reduction, as measured with the Sleep Reduction Screening Questionnaire and additional sleep diary questions (SRSQ) [92].

##### Adolescents in the E-TRAIN group

- Intervention uptake of E-TRAIN, measured through log data of the online platform.
- Treatment satisfaction, as measured with a modified version of the Client Satisfaction Questionnaire (CSQ-8) [93].

##### Parent/ caregiver measures

Additional parent/ caregiver measures that will be assessed are:

- Socio-demographics of the parent, collected at baseline.
- Emotion regulation of the child of adolescent?, as measured with the FEEL-KJ – Parent Report (FEEL-KJ: Parent Report) [78, 94].
- Parental? Coping with the adolescent’s negative emotions, as measured with the Coping with Children’s Negative Emotions Scale – Adolescent Version (CCNES-A) [95].
- Emotion dysregulation of the parent, as measured with the Difficulties in Emotion Regulation Scale (DERS) [79].
- Psychiatric distress of the parent, as measured with the Brief Symptom Inventory – short version (BSI-18) [96].

##### Therapist measures

The CBT therapist of each participant will be asked to report the following: number of CBT sessions, content and number of sessions of non-CBT care. Additionally, the E-TRAIN therapist will be asked to fill in an adapted version of the System Usability Scale to measure the usability of the online intervention (SUS) [97].

### Data analyses

#### Primary outcome measures

Linear mixed models will be used to determine the effectiveness of adding E-TRAIN to CBT in reducing depressive and anxiety symptoms (measured with the RCADS25). A two-level structure will be used (Level 1: repeated measures, Level 2: patients). The overall treatment effect will first be evaluated in a model with condition and the baseline value of the outcome. Second, between-group differences at each time-point will be examined by adding time and the interaction of time and condition to the model. Time will be treated as a categorical variable, represented by dummy variables. Furthermore, as a sensitivity analysis, we will fit a model adjusted for the total number of completed CBT sessions and other potentially relevant confounders. Imputation of missing data will not be necessary because we will use mixed-model analyses, which adequately handles missing values [98]. Analyses will be conducted on the entire randomized sample (i.e. intention to treat) and on the per protocol/treatment completers sample. For the treatment completers sample of the experimental (E-TRAIN) condition, adolescents who completed ≥ 66% of the online sessions (four sessions or more) will be regarded as completers. The aforementioned main analyses will also be conducted separately for those with a primary diagnosis of depressive disorder, and for those with a primary diagnosis of anxiety disorder. Analyses will be conducted using SPSS version 26 + and R version 3.0 + . Statistical significance will be set at α < 0.05.

#### Secondary outcome measures

Linear mixed models will also be used to determine the effectiveness of the internet-based emotion regulation training with regard to 1) depressive symptoms (CDI-2); 2) anxiety symptoms (SCARED); 3) parent-report depressive and anxiety symptoms (SCARED-P and CDI-P); 4) therapist-report global functioning, symptom severity and improvement (CGAS and CGI); and 5) self-report emotion (dys)regulation (FEEL-KJ and DERS). Generalized linear mixed model analysis will be used to determine the effectiveness of the internet-based emotion regulation training on the presence of a depressive and/or anxiety disorder (MINI (KID)). The aforementioned procedures will be followed for these secondary analyses.

#### Acceptability and feasibility of E-TRAIN in adolescents

To examine the acceptability and feasibility of E-TRAIN in adolescents and therapists, qualitative research methods aimed at analyzing focus groups will be used.

### Data collection and management

Data collection and management will be conducted with use of Castor EDC. Castor EDC complies with all applicable laws and regulations: Good Clinical Practice (GCP), 21 CFR Part 11, EU Annex 11, and the European Data Protection Directive. Castor EDC servers are located in the Netherlands and hosted according to the international information security norm ISO 27001/27002/9001 and in accordance with the Dutch NEN 7510 norm for information security in healthcare. The E-TRAIN module will be available for participants on the online *Jouw Omgeving* platform: a widely used e-mental health platform for youths that meets all technical demands and guidelines with regard to privacy and data security, such as ISO 27001 and NEN 7510.

## Discussion

This paper describes the study protocol of a randomized controlled trial aimed at assessing the effectiveness of the addition of a guided internet-based emotion regulation training (E-TRAIN) to CBT in reducing depressive and anxiety symptoms in adolescents with a depressive or anxiety disorder compared to CBT alone. Given the high prevalence and undesirable effects of anxiety and depressive disorders in adolescents, it is of great importance to improve treatment outcomes of these patients. Dysfunctional emotion regulation appears to be an important target of intervention in adolescents with aforementioned disorders, and adolescence appears to be a critical time period to enhance emotion regulation strategies. To the best of our knowledge, this study is the first to examine the effectiveness of a guided online emotion regulation intervention in adolescents with a depressive or anxiety disorder. We will investigate whether treatment outcome of CBT may be improved by strengthening ER skills in this vulnerable population. Moreover, this study will enhance knowledge on ER and its associated factors in adolescents with depression and anxiety.

Strengths of the current study are its randomized design and the relatively long follow-up period of 12 months. Another strength is the large battery of clinically relevant and validated outcome measures that are administered by multiple informants at multiple assessments. An important concern in the field of e-health research is treatment adherence, since internet-based treatments generally are associated with high dropout levels (e.g., [47]). It is expected that the relatively small amount of E-TRAIN sessions, three video conference sessions and weekly guidance of the therapist will enhance treatment adherence. However, this will remain an important challenge of this trial.

If this study provides evidence for the effectiveness of E-TRAIN, it can be an important addition to mental health care for adolescents with depressive and/or anxiety disorder.

## Supplementary Information

**Additional file 1. **English translation of the self-developed COVID-19 Questionnaire.

**Additional file 2. **English translation of the self-developed Life Events Questionnaire.

## Data Availability

During the ongoing data collection researchers will only have restricted access to the data. After completion of the study, only the Principal Investigator (CC) and the Study Coordinators (JE and MW) will have access to the data. Raw data cannot be made openly accessible to the public, due to privacy concerns. After request to the corresponding author, the data, statistical parameters and statistical code will be made accessible. Results will be published in international peer-reviewed journals and will be presented at scientific conferences. We will also report our results to relevant stakeholders in newsletters and trial summaries. All participants and parents will receive a written summary of the results after completion of the study. The publication of the results will be independent of the results, whether or not the experimental intervention is effective. Eligibility of authorship will be decided according to the guidelines of the International Committee of Medical Journal Editors (ICMJE). In accordance with the national regulations in The Netherlands, data will be stored for 15 years.

## Abbreviations

AUDIT: Alcohol Use Disorders Identification Test
BSI-18: Brief Symptom Inventory – Short version
CBT: Cognitive behavioral therapy
CCNES-A: Coping with Children’s Negative Emotions Scale – Adolescent Version
CDI: Children’s Depression Inventory
CDI-P: Children’s Depression Inventory – Parent version
CGAS: Children’s Global Assessment Scale
CGI: Clinical Global Impression Scales
CSQ-8: Client Satisfaction Questionnaire
DERS: Difficulties in Emotion Regulation Scale
ER: Emotion regulation
FEEL-KJ: Der Fragebogen zur Erhebung der Emotionsregulation bei Kindern und Jugendlichen
IPPA: Inventory of Parent and Peer Attachment
MINI: Mini International Neuropsychiatric Interview
RCADS25: Revised Children’s Anxiety and Depression Scale – Short Version
RSES: Rosenberg Self-Esteem Scale
SCARED: Screen for Child Anxiety Related Disorders
SCARED-P: Screen for Child Anxiety Related Disorders—Parent Version
SRSQ: Sleep Reduction Screening Questionnaire
SUS: System Usability Scale
