# Supplementary material for: Effectiveness of an add-on guided internet-based emotion regulation training (E-TRAIN) in adolescents with depressive and/or anxiety disorders: study protocol for a multicenter randomized controlled trial
Source: BMC Psychiatry. 2022 Oct 14;22:646. doi: 10.1186/s12888-022-04291-6 (PMC9568959; doi:10.1186/s12888-022-04291-6)
Supplement: Supplementary file 1 — Additional file 1. English translation of the self-developed COVID-19 Questionnaire. [file 12888_2022_4291_MOESM1_ESM.pdf]

COVID-19 questionnaire

The following questions are specifically about how the COVID-19 and the COVID-19 restrictions have affected you. Please indicate to what extent this reflects your opinion regarding the past three months in the statements below.

1 = Strongly disagree
2 = Disagree
3 = Neutral
4 = Agree
5 = Totally agree
6 = I have not had any depressive symptoms in the past 3 months/ I have not had any anxiety symptoms in the past 3 months/ I do not drink alcohol/ I am currently not in school and I am not studying

In the past 3 months, my depressive symptoms have been aggravated by the Coronavirus and/or the COVID-19 restrictions	1	2	3	4	5	6
In the past 3 months, my anxiety symptoms have been aggravated by the Coronavirus and/or the COVID-19 restrictions	1	2	3	4	5	6
In the past 3 months, I have been drinking more alcohol due to the Coronavirus and/or the COVID-19 restrictions	1	2	3	4	5	6
In the past 3 months, I have slept worse due to the Coronavirus and/or the COVID-19 restrictions	1	2	3	4	5	
In the past 3 months, I have had less contact with peers than I would have liked due to the Coronavirus and/or the COVID-19 restrictions	1	2	3	4	5	
In the past 3 months, my school/study performance has deteriorated due to the Coronavirus and/or the COVID-19 restrictions	1	2	3	4	5	6
