# Supplementary material for: Effectiveness of an add-on guided internet-based emotion regulation training (E-TRAIN) in adolescents with depressive and/or anxiety disorders: study protocol for a multicenter randomized controlled trial
Source: BMC Psychiatry. 2022 Oct 14;22:646. doi: 10.1186/s12888-022-04291-6 (PMC9568959; doi:10.1186/s12888-022-04291-6)
Supplement: Supplementary file 2 — Additional file 2. English translation of the self-developed Life Events Questionnaire. [file 12888_2022_4291_MOESM2_ESM.pdf]

Life Events Questionnaire

Below is a list of negative life events that can happen to people. Have you experienced these events in the past year and/or earlier in your life?

1: Never
2: In the past year
3: Earlier in my life

1. My parents are divorced

1	2	3

If 3:
How old were you when this happened? age: __________year

2. One of my parents became seriously physically ill

1	2	3

If 3:
How old were you when this happened? age: __________year

3. My brother or sister became seriously physically ill

1	2	3

If 3:
How old were you when this happened? age: __________year

4. I was seriously physically ill myself

1	2	3

If 3:
How old were you when this happened? age: __________year

5. I have had a serious accident

1	2	3

If 3:
How old were you when this happened? age: __________year

6. One of my parents/caregivers has passed away

1	2	3

If 3:
How old were you when this happened? age: __________year

7. Someone else who is very important to me has passed away

1	2	3

If 2 or 3:
Which important person has passed away?_____________________________

If 3:
How old were you when this happened? age: __________year

8. One of my parents/caregivers had serious mental health problems

1	2	3

If 3:
How old were you when this happened? age: __________year

9. I have lived somewhere other than with my biological parents for a short or long time (for example, in a foster family, a children's home, or institution).

1	2	3

If 2 or 3:
This was:

- ☐ With adoptive parents
- ☐ In a foster family
- ☐ In a children's home
- ☐ In a (youth) institution
- ☐ Other: ________________

How long did this period last?

________ year(s) and ________ months

If 3:
How old were you when this happened? age: __________year

10. I have been physically abused (e.g. beaten, kicked, bitten, attacked, or beaten up)

1	2	3

If 3:
How old were you when this happened? age: __________year

11. Someone has threatened to harm me physically (without it actually happening)

1	2	3

If 3:
How old were you when this happened? age: __________year

12. I have been touched or held in a sexual way against my will

1	2	3

If 3:
How old were you when this happened? age: __________year

13. I believe I have been sexually abused

1	2	3

If 3:
How old were you when this happened? age: __________year

14. I have been verbally abused, insulted, bullied or threatened by one of my parents/caregivers

1	2	3

If 2 or 3: Did this happen multiple times?

☐ Yes

☐ No

If 3:
How old where you when this happened? age: __________year

15. I have been bullied by someone other than my parents/caregivers

1	2	3

If 2 or 3: Did this happen multiple times?

□ Yes

□ No

If 3:
How old were you when this happened? age: __________year

16. I did not have enough to eat at home, had to wear dirty clothes, or was left alone for days without someone to look after me

1	2	3

If 3:
How old were you when this happened? age: __________year

17. I usually could not turn to my parents/caregivers for the attention and support I needed, my parents/caregivers often did not listen to me or ignored me

1	2	3

If 3:
How old were you when this happened? age: __________year

18. I have witnessed my parents/caregivers use violence against each other or against my siblings (e.g. hitting, kicking, or throwing things at each other)

1	2	3

If 3:
How old were you when this happened? age: __________year

19. My parent/caregiver regularly used drugs or too much alcohol when I lived with him/her

1	2	3

If 3:
How old were you when this happened? age: __________year

20. I have been stalked or severely harassed by someone over the phone or on the internet.

1	2	3

If 3:
How old were you when this happened? age: __________year

21. I have lived in a place where there was a war/where there was a lot of fighting in the streets

1	2	3

If 3:
How old were you when this happened? age: __________year

22. I have experienced another very stressful life event, which is:

____

____

1	2	3

If 3:
How old were you when this happened? age: __________year
